# Remarks on Methylene

**Published:** 1873-11

**Authors:** 


### REMARKS ON METHYLENE.

Dr. Richardson says that this anæsthetic is rapid in its effect, that "from one to two drachms will induce, in the space of a minute and a half to two minutes, sufficient insensibility for a short operation, while from two to six drachms are sufficient for the production of prolonged anæsthetic sleep." The sleep induced is very gentle, and rarely attended with convulsive movement; vomiting is less frequent than from chloroform, ether, or bichloride of methylene.—*Med. and Surg. Reporter.*

